# Understanding autism in the light of sex/gender

**DOI:** 10.1186/s13229-015-0021-4

**Published:** 2015-05-13

**Authors:** Meng-Chuan Lai, Simon Baron-Cohen, Joseph D Buxbaum

**Affiliations:** Autism Research Centre, Department of Psychiatry, University of Cambridge, Douglas House, 18B, Trumpington Road, Cambridge, CB2 8AH UK; CLASS Clinic, Cambridgeshire and Peterborough NHS Foundation Trust, Chitra Sethia Autism Centre, The Gatehouse, Fulbourn Hospital, Fulbourn, Cambridge, CB21 5EF UK; Department of Psychiatry, National Taiwan University Hospital and College of Medicine, No.1 Jen-Ai Road Section 1, Taipei, 10051 Taiwan; Seaver Autism Center for Research and Treatment, Departments of Psychiatry, Neuroscience, and Genetics and Genomic Sciences, Friedman Brain Institute and Mindich Child Health and Development Institute, Icahn School of Medicine at Mount Sinai, 1468 Madison Avenue, New York, NY 10029 USA

## Editorial

Autism has long been described as a predominantly male condition, with a widely cited male:female ratio of 4 to 5:1 [1]. Over the past two decades, we have witnessed a trend towards a decreasing male-predominance [2]. Latest large-scale population/community-based epidemiological studies converge to show a ratio of 2 to 3:1 [3]. The implications of this are important.

Females with autism may have been underidentified and therefore underrepresented in the past, and consequently, the previous scientific and clinical literature likely may have provided a male-biased understanding of autism. However, a relative male-predominance remains a stable observation over time, which has led to fruitful aetiological and developmental investigations and theories [4-7,3]. For the field to move towards an integrated understanding of the whole autism spectrum, it is important that females are not overlooked, and their particular experiences are properly documented [8]. Sex and gender provide unique angles for understanding causal mechanisms in atypical human developmental conditions [9] and should be a central theme in the understanding to autism and its vast heterogeneity [3,10].

Note that the term ‘sex’ refers to ‘the biological and physiological characteristics that define men and women’, and ‘gender’ refers to ‘the socially constructed roles, behaviors, activities, and attributes that a given society considers appropriate for men and women’ [11]. Since many human studies of autism focus on children, adolescents and adults, it is often difficult to empirically separate the effect of sex and gender, since gendered socialization begins at birth. Therefore, unless when we specifically refer to ‘sex’ or ‘gender’ separately, as defined here, we use the term ‘sex/gender’ to note the inevitable overlap between them [12].

*Molecular Autism*, since its launch in 2010, has published a range of research in relation to sex/gender and autism, for example, about male-female differences and female-specific characteristics of autism [13-15]; issues related to potentially ‘missed’ females on the spectrum, diagnostic overshadowing and co-occurrence [16-19]; sex/gender-differential traits in the general population in relation to the liability of developing autism [20,21]; and biological aetiologies associated with sex-differential liability [22-28].

Acknowledging the need to bring sex and gender to centre stage of autism research, in 2014, we launched a call-for-papers for the inaugural thematic series of this journal, ‘Understanding the links between sex/gender and autism.’ This thematic series are comprised of two batches of research articles, with the first being published in May 2015 [29-33], followed by a second later in 2015.

It has recently been proposed that research into sex/gender and autism could benefit from adapting a conceptual framework including four separate but interlinked themes: (1) nosological and diagnostic challenges, (2) sex/gender-independent and sex/gender-dependent characteristics, (3) general models of aetiology regarding liability and threshold, and (4) specific aetiological-developmental mechanisms [3]. The five articles in this issue cover all of these four major themes, providing novel and important findings.

Addressing aspects of the first theme and building on recent investigations into the co-occurrence of increased autistic traits [16-18] and autism diagnoses [34] in individuals with eating disorders, Mandy and Tchanturia’s case series [33] report that in ten women with eating disorders and social/flexibility difficulties, seven were judged to have an autism spectrum disorder (ASD) by gold-standard diagnostic procedures (particularly including in-person observation using the Autism Diagnostic Observation Schedule). Although all seven women with ASD presented difficulties closely related to autism early in life and prior to their eating disorder diagnoses, only one had received an ASD diagnosis in childhood. Findings from a referred case series may not always reflect what is happening in community or family clinics or general mental health services; however, Mandy and Tchanturia raise the alarm in relation to potential under-/misdiagnosis of autism or diagnostic ‘overshadowing’ in females with autism with an eating disorder. They do this by employing formal diagnostic procedures, rather than merely measuring self-reported dispositional traits.

A similar point is made through case series of other kinds of psychopathology [35-37]. Females with autism tend to be identified later than males [38-40], and diagnoses tend to be given when autistic characteristics or concurrent behavioural/cognitive difficulties are more severe [41,42]. Although women (as well as men) diagnosed with autism have been shown to have high rates of concurrent psychiatric diagnoses [43,44], it is unknown whether females are more likely than males to first get other psychiatric diagnoses (either truly comorbid or misidentified) before the identification of their autism. How diagnostic overshadowing, substitution or co-occurrence influences the timing and likelihood of the identification of autism in females (and the associated need for support) should be an important focus for future epidemiological, clinical and health-care research [3].

Identifying similarities and differences between males and females with autism across levels has the potential to inform both identification (for example, behavioural features) and understanding of aetiologies (for example, biological features). Recent investigations have considered aspects of cognition and biology and suggest that, contrary to the conventional view that diagnosed females tend to be ‘more severe’, they may rather have ‘different’ biological characteristics compared to males with autism (beyond preexisting normative sex/gender differences); see [45,3] for reviews. Nordahl *et al*. [32] extend this by showing that there are sex/gender differences in the pattern of altered corpus callosum neuroanatomy in a longitudinal sample of preschooler with autism. They found males and females with autism, compared to same-sex/gender controls, differ in different callosal subregion volume (classified based on cortical projection zone) and microstructural properties. Interestingly, no sex/gender differences in growth trajectory were discovered, suggesting that the observed sex/gender-dependent white matter characteristics were established prior to age of three or that the age window of this study (3 to 5 year-olds) reflected a period without evident sex/gender-differential growth patterns. Together with other recent findings on very early sex/gender differences in brain and physical growth [46-48], this suggests male-female biological differences are evident early in life in autism, possibly reflecting the key role of early biological factors giving rise to sex-differential aetiological mechanisms, such as prenatal steroids [49] and associated regulatory mechanisms [29] or early neuroinflammatory mechanisms [50].

Two articles further examine aetiological implications, focusing on the ‘female-protective effect’ (FPE) hypothesis. Werling and Geschwind [30] use a large sample of multiplex families and twin pairs in the Autism Genetics Resource Exchange (AGRE) cohort to test two predictions from the FPE hypothesis: first, that males show higher rates of autism than females (prediction 1), and second, that risk of autism is greater for the siblings and co-twins of females with autism than siblings and co-twins of males with autism (prediction 2). Prediction 1 has been repeatedly confirmed across studies (that is, the relative male-predominance in prevalence/incidence). Prediction 2 has been supported from general population twin samples by measuring autistic traits [51], yet when examining clinical autism diagnoses, the results are inconsistent and are even negative in some large-scale general population studies [52,53]. Here, Werling and Geschwind show that in multiplex families both predictions are confirmed, using a less biased yet more conservative approach than previous studies (that is, using two probands instead of one to define ‘male-only’ (MO) versus ‘female-containing’ (FC) families, preventing artifactual inflation of recurrence rates in FC families and deflation in MO families).

The contrast between this confirmation of prediction 2 and previous negative general population findings might be due to differences in ascertainment or sensitivity to diagnosis, particularly in females (for example, better sensitivity to detecting autism in females in multiplex families than in general community clinical settings). A further interesting observation is that prediction 1 reflecting FPE (male > female relative risk, RR = 2.25) is more evident and substantial than prediction 2 (sibling in FC > sibling in MO families, RR = 1.46). Although both predictions are thought to reflect FPE, mechanisms leading to prediction 2 may be more complicated. For example, the reason why females are generally less likely to have autism (prediction 1) may be the operation of ubiquitous genetic and/or environmental *protective* mechanisms to females rather than males. However, reasons causing increased RR in siblings in FC than siblings in MO families (prediction 2) may be contributed further by other *risk* mechanisms (for example, increased inherited mutations occurring in previous generations and ‘carried’ by unaffected/undiagnosed females in the family [54]) that act against the ubiquitous female-protective mechanisms. For prediction 2 to be observed, ubiquitous female-protective effects have to be overwhelmed by additional risk mechanisms. Elucidating the different mechanisms/factors contributing to FPE should be a focus of aetiological investigation.

Gockley *et al*. [31] performed a female-only genome-wide association study (GWAS) on the AGRE cohort (with replication using the Simons Simplex Collection (SSC) data). Based on the observation of a bimodal distribution of autistic traits in AGRE female but not male participants, they hypothesised that FPE could be mediated by a single, common genetic locus on chromosome X, an obvious candidate for protection to females (that is, aspects of FPE reflected by prediction 1). They concluded that despite the analysis was well powered, no evidence of a common variant accounting for a single FPE locus was observed; however, the possibility of multiple genetic loci contributing to FPE is not excluded. It might be informative to interpret the findings in the light of the different aspects of FPE mentioned above. Genetic markers revealed by a female-only GWAS comparing females with and without autism may be more associated with risk mechanisms that act against and overwhelm ubiquitous female-protective mechanisms, as the comparison is between those being successfully protected (unaffected females) and those affected despite being equally protected (females with autism). In other words, what Gockley *et al*. showed is probably beyond the associative factors for prediction 1 but more about prediction 2 (that is, risk mechanisms acting against protection). As the authors pointed out in their discussion, mechanisms for FPE are likely to be multi-factorial and may involve both genetic and environmental factors.

There are some other interesting findings revealed by these two aetiological investigations. Contrary to the conventional impression of greater male skew amongst more cognitively able individuals, Werling and Geschwind found in their AGRE sample the male-female ratio is actually smaller at the ‘higher-functioning’ end, echoing similar recent epidemiological findings [55,56]. These altogether challenge the long-held view that diagnosed females tend to be more ‘severely affected’ than males (that has been used to formulate and test the multi-factorial liability models of autism aetiology [5,57]) and point to the need to reevaluating the liability models and FPE considering sex/gender-dependent mechanisms [3]. Gockley *et al*., in interpreting their findings, discussed potential environmental factors contributing to FPE; Werling and Geschwind also suggested possibly greater environmental aetiological contribution in males in interpreting the male-specific association between shorter inter-birth intervals and increased autism recurrent risk. Nongenetic (likely including environment and gene-environment interplay) contributions to the emergence of autism have been highlighted recently [58,53,59-61]. It would be informative to further consider how sex/gender moderates gene-environment mechanisms of autism, such as through sex-specific multiple ‘hits’ [7].

Most of the aetiological investigations start from phenotypic observations. Sex/gender-related nosological and identification challenges however still receive insufficient examination, potentially biasing aetiological inferences [3]. For example, the bimodal distribution of SRS scores in AGRE females might partially stem from ascertainment characteristics, as Gockley *et al*. discussed, but it could also partially stem from ‘polarised’ rating for females by informants, influenced by existing gender stereotypes (that is, rating affected females as more severe because the characteristics are perceived as more deviant from the general population ‘female norm’ and stereotype whereas rating unaffected females as having even fewer autistic characteristics because they are perceived by the informants as better fitting the typical female norm and stereotype, given the contrast by the presence of affected probands in the family). More discussion into this challenge will be beneficial for advancing our understanding to how sex/gender interacts with aetiological mechanisms in autism.

Finally, Hu *et al*. [29] look into specific sex-implicated aetiological mechanisms. Their previous studies show that autism may be associated with deficiency in the expression of the retinoic acid-related orphan receptor alpha (RORA) gene, and therefore, dysregulated feedback loops with androgen and oestrogen, as well as the many autism-associated genes regulated by RORA [62,23,22]. In their new article, Hu *et al*. show that there are sex differences in RORA expression in specific brain regions in both human and mice and in the correlation between RORA and its transcriptional targets. This suggests that, at least in a subgroup of autism, males and females may be differentially affected by dysregulated RORA expression. Specifically in their mice model, disruption of RORA seems to have a more substantial effect in males than females. Although this preliminary study awaits further replication in larger samples, it shows that some aetiological mechanisms of autism could be sex-dependent and involve recursive, multi-level pathways.

More importantly, this has to be delineated in the context of gene-environment interplay. For example, the potential aetiological role of RORA deficiency and prenatal hormonal effects could be an example of gene-environment correlation in which biological sex plays a moderating role. Recent epidemiologically based endocrine findings show increased prenatal steroidogenic activity being an early ‘environmental’ risk for later autism diagnosis in males [49]. This could be further dissected in the context of gene-environment interplay. Male-specific risks by environmental factors and gene-environment interplay as suggested by both Hu *et al*. and Werling and Geschwind, and more generally by the well-known findings of increased susceptibility to hazard in males than females during foetal periods, should be a focus of aetiological investigation for autism.

Autism should not be perceived as a ‘male condition’. Females should no longer be underrepresented in future autism research. Unanswered questions span issues from definition, identification, presentation and characteristics to aetiological and developmental mechanisms, and these will be better understood in the light of sex and gender.

We are grateful to all the authors contributing to part 1 of this thematic series, who join forces to elucidate the complex but informative relationships surrounding sex/gender and autism. We hope this will stimulate more research into this topic.
